# Expert consensus and recommendations on safety criteria for active mobilization of mechanically ventilated critically ill adults

**DOI:** 10.1186/s13054-014-0658-y

**Published:** 2014-12-04

**Authors:** Carol L Hodgson, Kathy Stiller, Dale M Needham, Claire J Tipping, Megan Harrold, Claire E Baldwin, Scott Bradley, Sue Berney, Lawrence R Caruana, Doug Elliott, Margot Green, Kimberley Haines, Alisa M Higgins, Kirsi-Maija Kaukonen, Isabel Anne Leditschke, Marc R Nickels, Jennifer Paratz, Shane Patman, Elizabeth H Skinner, Paul J Young, Jennifer M Zanni, Linda Denehy, Steven A Webb

**Affiliations:** Australian and New Zealand Intensive Care Research Centre (ANZIC-RC), Department of Epidemiology and Preventive Medicine, School of Public Health and Preventive Medicine, Monash University, 99 Commercial Road, Melbourne, Victoria 3004 Australia; Department of Physiotherapy, The Alfred, 55 Commercial Road, Melbourne, Victoria 3004 Australia; Department of Physiotherapy, Royal Adelaide Hospital, North Terrace, Adelaide, South Australia 5000 Australia; Outcomes After Critical Illness & Surgery (OACIS) Group, Division of Pulmonary & Critical Care Medicine, Department of Physical Medicine & Rehabilitation, Johns Hopkins Hospital, 600 N. Wolfe Street, Meyer 1-130, Baltimore, Maryland 21287 USA; School of Physiotherapy & Exercise Science, Curtin University of Technology, Kent Street, Bentley, Western Australia 6102 Australia; School of Health Sciences, University of South Australia, City East Campus, North Terrace, Adelaide, South Australia 5001 Australia; Physiotherapy Department, Flinders Medical Centre, Flinders Drive, Bedford Park, South Australia 5042 Australia; Physiotherapy Department, Austin Hospital, 145 Studley Road, Heidelberg, Victoria 3084 Australia; The Prince Charles Hospital, 627 Rode Road, Chermside, Queensland 4032 Australia; Faculty of Health, University of Technology, Sydney (UTS), 235 Jones Street, Broadway, New South Wales 2007 Australia; Physiotherapy Department, Canberra Hospital, Yamba Drive, Garran, Australian Capital Territory 2605 Australia; Department of Physiotherapy, St. Vincent’s Hospital Melbourne, 41 Victoria Parade, Fitzroy, Victoria 3065 Australia; Department of Anaesthesiology and Intensive Care, Töölö Hospital, Helsinki University Central Hospital, Topeliuksenkatu 5, Helsinki, Finland; Intensive Care Unit, Canberra Hospital, Yamba Drive, Garran, Australian Capital Territory 2605 Australia; Australian National University Medical School, The Canberra Hospital, Hospital Road, Garran, Australian Capital Territory 2606 Australia; Physiotherapy Department, Princess Alexandra Hospital, 199 Ipswich Road, Woolloongabba, Queensland 4102 Australia; Burns, Trauma & Critical Care Research Centre, School of Medicine, The University of Queensland, Department of Intensive Care Medicine, Royal Brisbane and Women’s Hospital, Herston, Queensland 4029 Australia; School of Allied Health Sciences, Griffith University, Gold Coast Campus, Parklands Drive, Southport, Queensland 4215 Australia; School of Physiotherapy, The University of Notre Dame Australia, Fremantle Campus, 19 Mouat Street, Fremantle, Western Australia 6959 Australia; Department of Physiotherapy, Western Health, Western Hospital, Gordon Street, Footscray, Victoria 3011 Australia; Department of Physiotherapy, School of Primary Health Care, Monash University, McMahons Road, Frankston, Victoria 3199 Australia; Capital and Coast District Health Board, Intensive Care Unit, Wellington Hospital, Riddiford Street, Wellington, 6021 New Zealand; Medical Research Institute of New Zealand, Wellington Hospital, Riddiford Street, Wellington, 6021 New Zealand; Department of Physical Medicine and Rehabilitation, Johns Hopkins Hospital, 600 N. Wolfe Street, Meyer 1-130, Baltimore, Maryland 21287 USA; Department of Physiotherapy, Melbourne School of Health Sciences, The University of Melbourne, Parkville, Victoria 3010 Australia; School of Medicine and Pharmacology, The University of Western Australia, 35 Stirling Highway, Crawley, Perth, Western Australia 6006 Australia

## Abstract

**Introduction:**

The aim of this study was to develop consensus recommendations on safety parameters for mobilizing adult, mechanically ventilated, intensive care unit (ICU) patients.

**Methods:**

A systematic literature review was followed by a meeting of 23 multidisciplinary ICU experts to seek consensus regarding the safe mobilization of mechanically ventilated patients.

**Results:**

Safety considerations were summarized in four categories: respiratory, cardiovascular, neurological and other. Consensus was achieved on all criteria for safe mobilization, with the exception being levels of vasoactive agents. Intubation via an endotracheal tube was not a contraindication to early mobilization and a fraction of inspired oxygen less than 0.6 with a percutaneous oxygen saturation more than 90% and a respiratory rate less than 30 breaths/minute were considered safe criteria for in- and out-of-bed mobilization if there were no other contraindications. At an international meeting, 94 multidisciplinary ICU clinicians concurred with the proposed recommendations.

**Conclusion:**

Consensus recommendations regarding safety criteria for mobilization of adult, mechanically ventilated patients in the ICU have the potential to guide ICU rehabilitation whilst minimizing the risk of adverse events.

## Introduction

In the past, critically ill patients who were receiving mechanical ventilation were often managed with deep sedation and bed rest, at least during the early stages of their ICU admission. Despite long-standing evidence that prolonged bed rest results in deconditioning [1,2], studies investigating the effectiveness of early progressive mobilization for ICU patients have only started appearing in the literature in the last 10 to 15 years [3,4]. While the earlier publications documented the feasibility, safety and physiological effects associated with the mobilization of ICU patients [5-8], point-prevalence studies [9,10] and controlled trials investigating the effectiveness of early progressive mobilization have been published in more recent years [11-16]. These studies, and concomitant systematic reviews [4,17-22], provide evidence that early progressive mobilization of adult ICU patients is feasible, safe, and may result in benefits including improved functional outcomes, and reduced ICU and hospital length of stay.

These findings are contributing to a shift in ICU clinical practice, where patients who once would have received deep sedation and bed rest, are now less heavily sedated and receive early progressive mobilization [23]. The incidence of reported adverse events associated with early progressive mobilization of ICU patients is low (≤4%) [17]. Moreover, most of these adverse events were transient and benign. Whilst it is important that consideration is given to the potential benefits versus the potential adverse events associated with early progressive mobilization, it is possible that undue concern about adverse events may be resulting in mobilization being withheld where it might otherwise be beneficial. In order for early progressive mobilization to be undertaken safely in an ICU setting, with a minimal risk of adverse sequelae, it is essential that patients be carefully assessed prior to any mobilization intervention. Such assessment is facilitated by the availability of objective criteria that indicate that it is reasonable or safe to initiate mobilization [24]. A logical process for the development of such criteria is to utilize expert opinion to achieve consensus and, subsequently, determine the validity of these criteria by empiric research. The aim of this study was to develop consensus recommendations on safety criteria that should be considered prior to mobilizing adult, mechanically ventilated, ICU patients.

## Methods

A group of 23 multidisciplinary experts who had considerable clinical experience and were currently involved in research about early mobilization of adult ICU patients were invited to participate in a consensus meeting. All participants were based at tertiary centers. All 23 invitees attended a face-to-face meeting on 21 June 2013. These 23 participants comprised 17 physiotherapists, 5 intensivists and 1 nurse, who were from Australia (n = 19), United States (n = 2), New Zealand (n = 1) and Finland (n = 1).

Prior to the face-to-face meeting, a systematic review of the literature was performed by two members of the group (CH, CT). Protocols and publications that outlined safety criteria for early mobilization in ICU were identified and distributed to the group. Additionally, any publication or protocol that a member of the consensus committee deemed important was circulated prior to the meeting.

The face-to-face meeting was divided into three parts. First, there were presentations from individual panel members of any published or unpublished safety criteria for mobilization. Second, the panel members were divided into small working groups to determine where there was clear agreement and where further discussion was required regarding safety criteria. Third, the entire group then re-formed and discussed the recommendations from the smaller working parties in order to determine where consensus had been reached and where further discussion was required. Following the face-to-face meeting, a summary of the safety criteria for mobilization was drafted and, using an iterative process, was circulated to panel members via email until the group had reached consensus or agreed that they could not reach consensus. Consensus was defined as 100% agreement amongst the group.

## Results

### Nature of the safety recommendations

The consensus group agreed that the recommendations were aimed at assisting in the assessment of adult, mechanically ventilated ICU patients to determine if and when mobilization could commence. A critical element that was adopted was that these criteria should be regarded as a guide and should always be used in conjunction with clinical reasoning. It was agreed that the input into the decision to mobilize should lie with all members of the multidisciplinary team (that is, physiotherapy, medical, nursing staff) with the treating clinician having ultimate responsibility for decision making.

**Figure 1:**
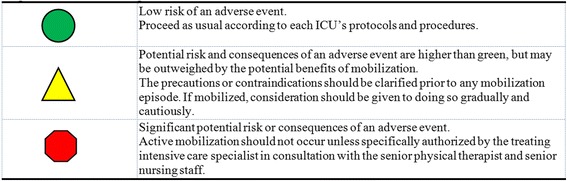
**Color coding definitions.**

The safety criteria developed by the group are intended to be used whenever mobilization is considered, which might be up to several times per day for an individual patient. The consensus group agreed that a standard traffic-light system of recommendations would be used to assist clinicians in evaluating safety criteria, where red would indicate the need for caution as the risk of an adverse event, or consequences of an adverse event, was high, yellow would indicate that mobilization was possible, but only after further consideration and/or further discussion among the ICU multidisciplinary team, and green would indicate that the patient was safe to be mobilized (see Figure 1). It was agreed that the most conservatively scored parameter must take precedence over all other scores (for example, a single red would be sufficient to caution about the potential for high risk of an adverse event during mobilization, even if all other parameters were green). In considering the decision to mobilize a patient, the criteria should be assessed on the status of the patient at the time of planned mobilization, but changes in condition, and direction of trends, in the preceding hours should also be taken into account. The potential consequences of an adverse event in an individual patient should also be considered as part of the overall clinical reasoning process.

The group decided that recommendations would be developed only for active mobilization and that no guidance would be provided with respect to safety criteria for passive mobilization. Active mobilization was defined as any activity where the patient assists with the activity using their own muscle strength and control: the patient may need assistance from staff or equipment, but they are actively participating in the exercise. Activities that comprise active mobilization are out-of-bed mobilization (that is, any activity where the patient sits over the edge of the bed (dangling), stands, walks, marches on the spot or sits out of bed) and in-bed mobilization (that is, any activity undertaken whilst the patient is sitting or lying in bed such as rolling, bridging, upper-limb weight training). The level of mobilization should be determined by the patient’s strength and endurance, as well as an assessment of the safety criteria.

The safety criteria covered by the consensus group were divided into four categories: (1) respiratory considerations, including intubation status, ventilatory parameters and the need for adjunctive therapies; (2) cardiovascular considerations, including the presence of devices, cardiac arrhythmias and blood pressure; (3) neurological considerations, including level of consciousness, delirium and intracranial pressure, and (4) other considerations, including lines and surgical or medical conditions.

### Respiratory safety considerations

**Figure 2:**
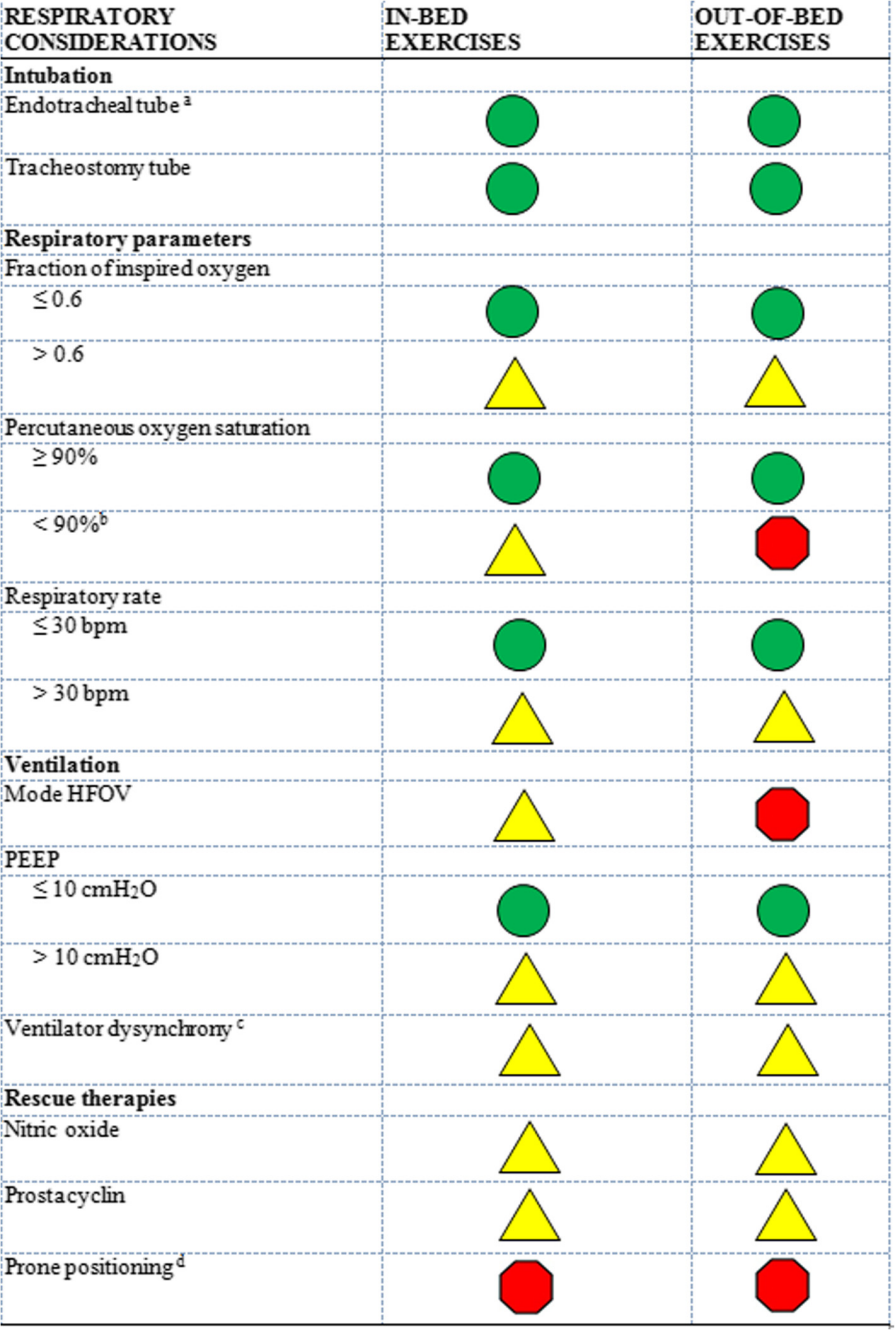
**Respiratory safety considerations.** PEEP, positive end-expiratory pressure.

Prior to each episode of mobilization, an appropriate healthcare professional, according to the procedures of each individual ICU, should check that any artificial airway present (that is, orotracheal, nasotracheal or tracheostomy tube) is correctly positioned and secure. Additionally, any supplemental oxygen that may be required by the patient should be available with an adequate oxygen reserve that exceeds the expected duration of the mobility activity (as unexpected delay or increased requirements may occur). The group agreed that endotracheal tube intubation was not in itself a contraindication to early mobilization and that a fraction of inspired oxygen (FiO2) less than 0.6 was a safe criterion for in- and out-of-bed mobilization if there were no other contraindications. Other respiratory safety recommendations are summarized in Figure 2. If the patient was at the safety limits for several categories (for example, low percutaneous oxygen saturation, high FiO_2_ and high positive end expiratory pressure), an experienced medical team should be consulted prior to mobilization.

### Cardiovascular safety considerations

**Figure 3:**
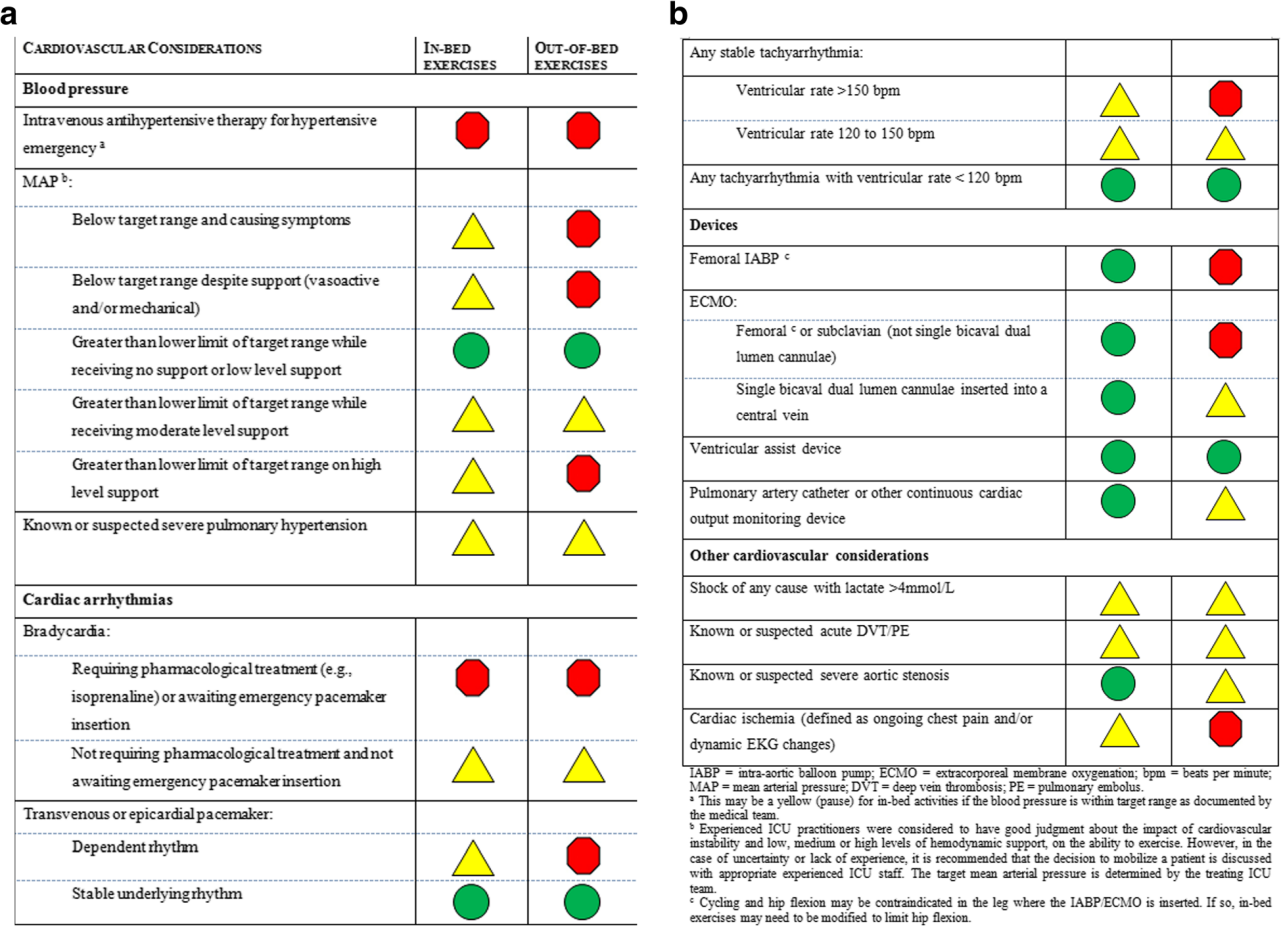
**Cardiovascular safety considerations.**

The cardiovascular considerations to be assessed prior to mobilization are summarized in Figure 3. Of note, panel members were unable to reach consensus regarding the dose of vasoactive drugs (and combination of these drugs) that would allow safe mobilization in the ICU setting; views on the dose, unit of measurement and combination of these drugs were variable across panel members of the consensus group. However, the group did reach consensus around the principles that were important to consider, which were that the administration of vasoactive drugs, *perse*, was not an absolute contraindication to mobilization but the appropriateness of mobilization was influenced by the absolute dose, the change in dose (for example, rising doses should result in caution or contraindication to mobilization), and, irrespective of the dose, whether or not the patient is clinically well-perfused. The group was unable to achieve consensus on a threshold dose of vasoactive medications below which it was acceptable to mobilize patients, the rate of change in dose and criteria for impaired perfusion and shock. It was therefore agreed that clinicians at individual ICUs should discuss the safe dose and combinations of vasoactive drugs that allows mobilization on a case-by-case basis with the appropriate ICU staff and that this represented a priority area for empiric research.

### Neurological and other safety considerations

**Figure 4:**
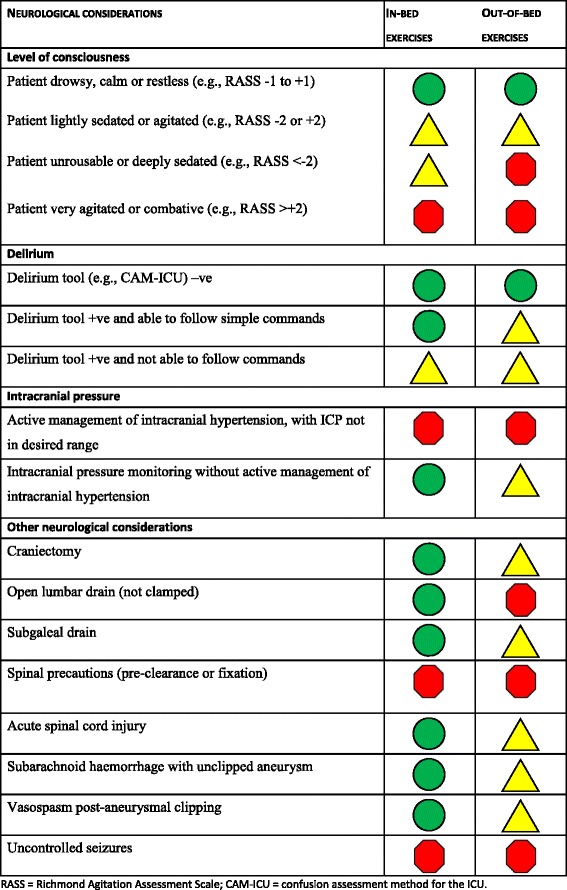
**Neurological safety considerations.** RASS, Richmond Agitation Assessment Scale; CAM-ICU, confusion assessment method for the ICU.

**Figure 5:**
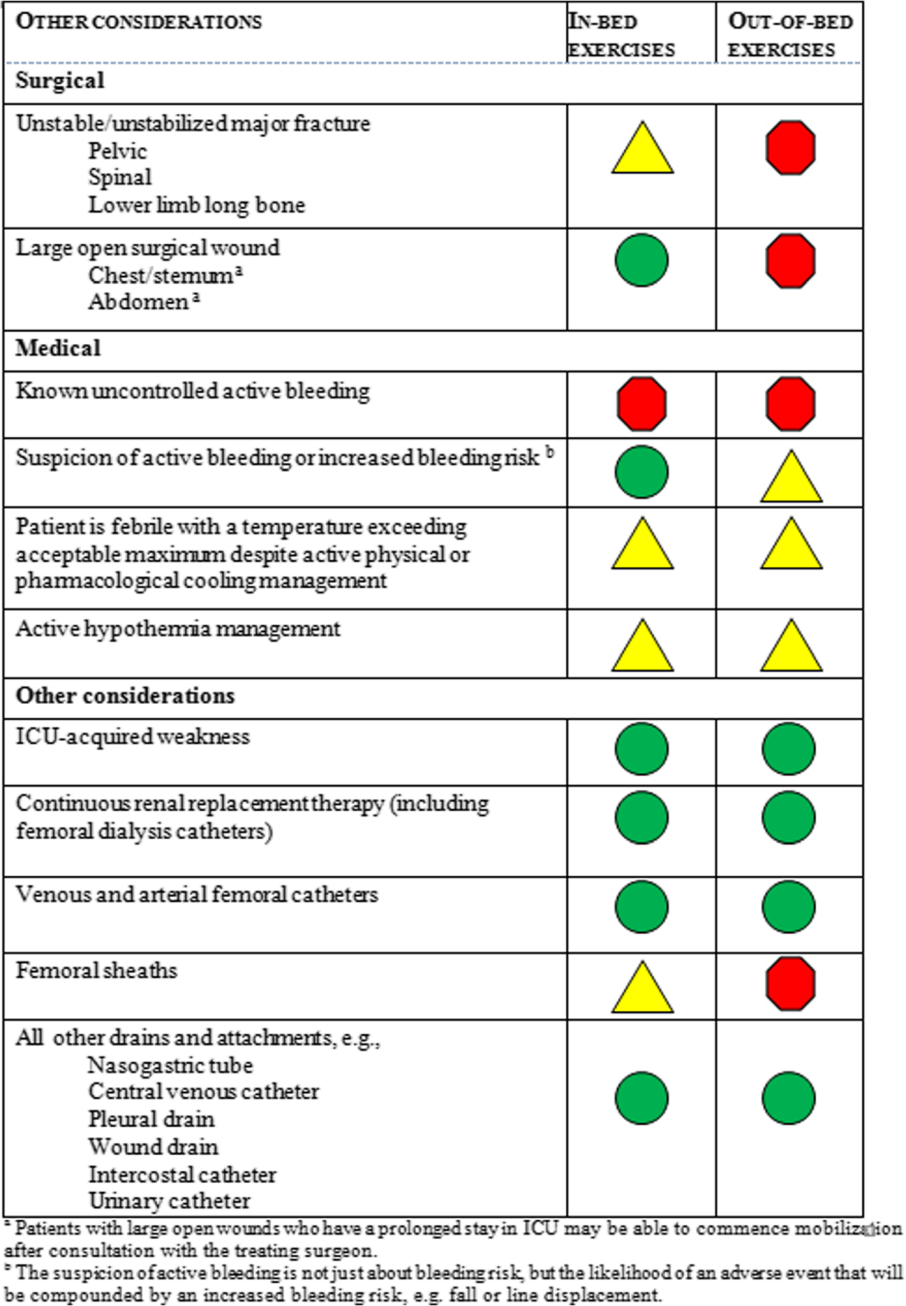
**Medical, surgical and other safety considerations.**

These are summarized in Figures 4 and 5 respectively.

## Discussion

The aim of this study was to develop consensus recommendations on safety criteria to determine readiness for actively mobilizing adult, mechanically ventilated, ICU patients. Utilizing previous evidence and expert opinion, the consensus group achieved consensus for most of the respiratory, cardiovascular, neurological and other safety considerations.

The criteria that have been used to determine when critically ill patients can be mobilized have varied between studies. Criteria for the early mobilization of adult ICU patients were published by Stiller and Phillips in 2004 [25], primarily based on physiological principles and their clinical experience, and were later endorsed by Gosselink *et al*. for the European Society of Intensive Care Medicine [26]. However, the level of evidence supporting these recommendations is limited. Compared to previous studies that have outlined safety parameters for the early mobilization of ICU patients, the recommendations outlined in this paper appear to be less conservative and more comprehensive by covering a wider array of clinical scenarios. The recommendations and clinical scenarios were identified by the group in an attempt to maximize mobilization of ICU patients. We believe these recommendations will assist in standardizing safety precautions regarding mobilization in ICUs across different healthcare centers and are appropriate for use by experienced ICU clinicians. However, each ICU should consider the recommendations in light of their own staffing levels and expertise. In the current study, panel members were unable to reach consensus for some safety considerations, specifically, the level of vasoactive drugs as noted earlier. Clearly, there is a need for research in this area to clarify safety parameters.

The strength of the safety recommendations outlined in this paper is that they are based on evidence from relevant clinical studies and required consensus of panel members, all of whom have clinical expertise and were currently involved in research regarding the early mobilization of ICU patients. There are several limitations to the current study as follows. The consensus group was predominantly comprised of clinicians working in Australia, therefore the recommendations may be reflective of Australian ICU culture and practice and thus may not be generalizable to other countries. However, the results of the consensus were presented at the Seventh International Meeting of Physical Medicine and Rehabilitation in Critically Ill held in San Diego on 17 May 2014. At this meeting there were 94 multidisciplinary clinicians, from both academic and non-academic hospitals, interested in early mobilization in ICU. Each of the criteria was discussed individually as documented and consensus was sought from attendees. Consensus was reached when 100% of attendees agreed to the proposed wording of the document. As a result of their feedback, minor amendments were made to the consensus document to reflect international practice. It is also acknowledged that the consensus recommendations are predominantly based on the experts’ interpretation of literature and their opinions which are based on their clinical practice.

Further research is required to validate each of the safety considerations discussed in these recommendations and the recommendations as a whole, both in centers with expertise in ICU mobilization and in centers without. Furthermore, as early progressive mobilization continues to be more extensively practiced and researched, and critical care medicine advances, it may be that criteria currently noted in red may become yellow in future versions of these recommendations. Finally, while the consensus group discussed safety parameters that should be assessed prior to mobilization, safety parameters that should be monitored during mobilization interventions were not considered.

## Conclusion

This study reports on the development of consensus recommendations outlining safety considerations prior to the mobilization of adult, mechanically ventilated patients in an ICU setting. The implementation of these recommendations has the potential to maximize early mobilization while minimizing the risk of adverse safety events, which in turn might improve functional outcomes and translate into reduced ICU and hospital length of stay. Future research required includes systematic evaluation of these recommendations.

## Key messages

- The safety criteria for mobilizing patients in ICU may be considered according to a traffic-light system of low risk of an adverse event (green), potential risk of an adverse event is outweighed by the benefit of early mobilization (yellow) and significant potential risk of an adverse event requiring consultation with senior ICU staff (red)
- The consensus for safe mobilization was provided for respiratory, cardiovascular, neurological and other considerations including lines and drains
- The group provided recommendations for active mobilization. No guidance was provided with respect to safety criteria for passive mobilization
